# Race/ethnic inequalities in early adolescent development in the United Kingdom and United States

**DOI:** 10.4054/demres.2019.40.6

**Published:** 2019-01-23

**Authors:** Afshin Zilanawala, Laia Bécares, Aprile Benner

**Affiliations:** 1 University College London (UCL), UK and Oregon State University, USA; 2 University of Sussex, UK; 3 University of Texas at Austin, USA

## Abstract

**BACKGROUND:**

Comparative literature investigating race/ethnic patterning of children’s health has found racial/ethnic minority status to be linked to health disadvantages. Less is known about differences during early adolescence, a period during which health outcomes are linked to later life health.

**OBJECTIVE:**

Using the UK Millennium Cohort Study (n = 10,188) and the US Early Childhood Longitudinal Survey–Kindergarten Cohort (n ~ 6,950), we examine differences in socioemotional and cognitive development among 11-year-old adolescents and the contribution of family resources in explaining any observed differences, including socioeconomic, cultural traditions, and psychosocial resources.

**RESULTS:**

Adverse socioemotional health and cognitive development were associated with race/ethnic minority status in both countries. In the United States, we found that cultural resources and family socioeconomic capital played a large role in attenuating differences in problem behaviors between Asian American, Black, and Latino adolescents and their White peers. In the United Kingdom, the explanatory factors explaining differences in problem behaviors varied by racial/ethnic group. In both contexts, family resources cannot explain the sizable cross-country differences in verbal skills. In the United Kingdom, Indian adolescents had nearly one-third of a standard deviation increase in their verbal scores whereas in the United States, Black and Latino adolescents had scores nearly two-fifths and one-fifth of a standard deviation below the mean, respectively.

**CONTRIBUTION:**

We use a detailed race/ethnic classification in the investigation of racial/ethnic inequalities across the United States and United Kingdom. There are strong family resource effects, suggesting that relative family advantages and disadvantages do have meaningful associations with adolescent socioemotional and cognitive development. Although levels of resources do explain some cross-national differences, there appears to be a broader range of family background variables in the United Kingdom that influence adolescent development. Our findings point to the critical role of both the extent and nature of family social capital in affecting adolescent development.

## 1. Introduction

A burgeoning line of comparative research examining race/ethnic inequalities in children’s health in the United Kingdom (UK) and the United States (US) has confirmed that, across national settings, racial/ethnic minority status is associated with a variety of markers of children’s health. Evidence of cross-national racial/ethnic inequalities have been found for low birth weight (Teitler et al. 2007), body mass index (Zilanawala et al. 2015a), and cognitive development (Washbrook et al. 2012), with disadvantages observed for children from Black and Asian aggregate ethnic groups in the United Kingdom, and for Hispanic, American Indian, and Black children in the United States, when compared to White British/American children. To date, however, no international comparative study has examined racial/ethnic inequalities in early adolescence, a particularly salient developmental period given the influence in both countries of a myriad of academic, social, and biological stressors (Benner and Wang 2015), including the need to achieve a sense of coherent identity and the necessity of navigating school transitions. In addition, much of the extant comparative literature on racial/ethnic disparities has focused on children’s physical health, and we know less about race/ethnic patterning in young people’s socioemotional health, an important predictor of academic achievement (Currie and Stabile 2007) and overall well-being.

To this end, we extend the current literature by using data from two comparable nationally representative cohort studies to examine racial/ethnic patterning in early adolescent socioemotional and cognitive development. As markers of health and development, we investigate early adolescent socioemotional well-being and cognitive development, which are strongly linked to education completion, labor force participation, and the formation of lifelong relationships (Patton et al. 2016). Socioemotional development is defined broadly as social, emotional, and behavioral problems, including externalizing and internalizing behaviors (Goodman 1997). Lastly, we examine the contribution of adolescent and family characteristics that could account for potential racial/ethnic differences in early adolescent development across sociodemographic, cultural, and psychosocial domains. By undertaking a cross-national, comparative approach, we are also able to determine whether the contributions of adolescent and family characteristics are similar across national settings.

### 1.1 Race/ethnicity differences in children’s socioemotional and cognitive development

Comparative literature on children’s mental health, child behavior, and cognitive development distinguishes differences between children of immigrant and native-born mothers instead of using detailed racial/ethnic classifications. Such crude categorizations have revealed inconsistent advantages and disadvantages. For example, in US and UK data examining 5-year-old children’s externalizing and internalizing behaviors, an immigrant advantage has been documented in externalizing behavior in the United States only, but immigrant disadvantages in internalizing behavior emerge in both the United States and United Kingdom (Jackson, Kiernan, and McLanahan 2012). Possible explanations focus on differing cultural beliefs and expectations between immigrants and natives and selection processes between families who migrate to the United States and United Kingdom versus those who remain in their home countries (Abraido-Lanza et al. 1999). A similar study investigating the same age group found no differences in child behavior between children of immigrant and native-born mothers in both contexts, but children of immigrants in the two countries performed more poorly on cognitive tests relative to their peers of native-born mothers (Washbrook et al. 2012). Parental language proficiency had important implications in explaining gaps between immigrant and native children.

Whereas comparative studies have been very useful in describing overall national patterns across countries, studies in the individual countries have provided greater detail on specific within-country differences across racial/ethnic groups. This work has emphasized that aggregations of racial/ethnic groups may obscure important differences in socioeconomic profiles and migratory histories, and potentially misattribute health advantages/disadvantages (Nazroo 2001; Zilanawala et al. 2015a). Indeed investigations of racial/ethnic inequalities in children’s health and development within each national context reveal a more complex narrative when using detailed racial/ethnic categories. In particular, in a sample of kindergarteners, children of Mexican origin had the lowest starting school readiness performance, whereas children of Asian origin outperformed their White peers in reading abilities in the United States, even after statistical adjustment for socioeconomic characteristics (Han, Lee, and Waldfogel 2012). Primary home language and parental English proficiency were highlighted as key family resources in accounting for differences in school readiness.

Relatedly, in a comprehensive analysis of a range of academic skills and behavior among US kindergarteners, Black and Hispanic children consistently underperform in math and reading skills, self-control, and approaches to learning (Reardon and Portilla 2016). By 5^th^ grade, more than half of Asian American children were performing at the highest levels for their group in reading achievement, whereas nearly three-quarters of African American children had reading skills that fell in the below average range (Davis-Kean and Jager 2014). The same study also found a considerable gap between high achieving Asian American children in math skills compared to their Hispanic and African American peers. In examining historical achievement gaps, a recent evaluation of the Black-White and Hispanic-White gaps in reading achievement found no evidence that gaps have narrowed for 9–10 year olds (Paschall, Gershoff, and Kuhfeld 2018). However, this study only examined one dimension of family resources, namely poverty status, to understand race/ethnic inequalities.

Turning to socioemotional health, Han, Lee and Waldfogel (2012) found fewer externalizing problems for Asian, Latino, and Mexican-origin children versus White children, more externalizing problems for Black versus White children, and fewer internalizing problems for other Asian and Mexican children versus White children. Moreover, during the elementary and middle school years, teachers and parents have consistently been found to rate African American children as having higher externalizing behavior than peers from other racial/ethnic groups (Keiley et al. 2000; Miner and Clarke-Stewart 2008).

In the UK context, studies using detailed race/ethnic categories reveal a mix of health disadvantages and advantages. Pakistani, Bangladeshi, and Black Caribbean 7-year-old children had more socioemotional difficulties than White children, and these differences were partially explained by the relative socioeconomic disadvantages of their families and the poor maternal mental health of racial/ethnic minority children (Zilanawala et al. 2015b). Black African children had fewer problem behaviors than White children, potentially due to the high labor force participation and educational attainment of their mothers. One recent UK study using repeated cross-sectional analysis found significant race/ethnic inequalities in verbal development in Indian, Pakistani, Bangladeshi, Black African, and Black Caribbean children up to 7 years of age, but the differences between race/ethnic minority children and their White peers diminished with increasing age (Smith, Kelly, and Nazroo 2015). In contrast, a study undertaking a longitudinal approach and including early adolescents (11 years old) found Pakistani and Bangladeshi children’s cognitive skills were consistently below average, and Indian children were above average across the first decade of life (ages 3 to 11), differences that were moderately explained by socioeconomic characteristics, cultural factors, and maternal mental health (Zilanawala, Kelly, and Sacker 2016).

UK studies focusing on adolescents suggest fewer socioemotional difficulties among most ethnic minority groups in the United Kingdom when compared to White children. For example, studies show an advantage for Indian and Pakistani children (Astell-Burt et al. 2012; Fagg et al. 2006; Goodman, Patel, and Leon 2010), Bangladeshi (Astell-Burt et al. 2012; Stansfeld et al. 2004) and Black African and Black Caribbean (Astell-Burt et al. 2012; Fagg et al. 2006) adolescents compared to their White peers in outcomes such as socioemotional health. However, such studies use wide age ranges, for example 5–15 years, making it difficult to disentangle whether more or fewer socioemotional difficulties emerge during adolescence, or if any ethnic variation exists during early adolescence. Given increased global migration and growing multicultural communities, we need a clearer picture of the level of psychosocial development and its associated risk factors among early adolescents across race/ethnic groups.

### 1.2 Comparative socioeconomic contexts for racial/ethnic group differences

In addition to the similarities in social and cultural contexts of United States and the United Kingdom, new availability of detailed comparable data facilitates the examination of cross-national comparisons in race/ethnic inequalities. Despite similarities across national contexts, it is also possible that the considerable heterogeneity in the composition of racial/ethnic groups in both countries may be reflected in relative differences in the outcomes of racial/ethnic minorities. Comparative studies have interrogated the causes of race/ethnic inequalities with a focus on the characteristics of the ethnic group itself (typically considered in terms of culture or genetics) and the social and economic disadvantages, the experience of migration, and the influence of racism faced by ethnic minority groups (Nazroo 2003). Before we can investigate causes or long-term consequences of racial/ethnic inequalities, we need to carefully evaluate the nature or extent of inequalities in socioemotional and cognitive development, and whether and how these differ across national settings, particularly during early adolescence. A comparative approach is useful for several reasons. Investigating racial/ethnic patterning in adolescent development in only one country may limit variation in social, historical, and economic factors, making it difficult to isolate pathways to positive versus negative outcomes for ethnic minority groups (Nazroo et al. 2007). The United Kingdom has a broader range of census categories for race/ethnic groups than those in the United States, and a comparative exercise may shed light on which patterns in the United States can and cannot be generalized to other ethnic subgroups and national settings. Although racial/ethnic groups in the United Kingdom (e.g., South Asians, Black African, and Caribbean groups) are distinctly different from those in the United States, in which migrants dominate from Mexican and Latin American origin, racial/ethnic minorities in both countries tend to have lower socioeconomic status (Zilanawala et al. 2015a), a similarity that would result in racial/ethnic minorities in both countries having worse developmental outcomes than their White counterparts. However, differences in the motivations and historical periods of immigration and forced migration across and within national contexts could lead to expected differences across developmental profiles across race/ethnic groups.

In the United States, European Americans have historically been the largest racial/ethnic group, alongside presenting with considerable socioeconomic advantages and institutional control (McDermott and Samson 2005). In contrast, African American and Hispanic groups have experienced significant discrimination and injustice. Considerable evidence underscores racial/ethnic gaps in achievement in the United States, with marked disadvantages for African American groups compared to their European American peers, and worryingly these gaps are increasing (Fryer and Levitt 2004). Conversely, Asian American groups have often been characterized as the ‘model minority’ and have lower risk of marginalization (Lee 2015); however, their access to power and prestige is not equal to that of European Americans (Kim 1999). Relative to their ethnic minority peers, Asian American children present with advantages in mathematics, language, and reading in early childhood (Farkas 2003).

Patterns of settlement and migration are very different in the United Kingdom because the presence of large numbers of racial/ethnic minorities is a relatively new phenomenon, following migration from Commonwealth countries in the 1950s and 1960s. More specifically, the primary wave of migration of Black Caribbeans and Indians to the United Kingdom occurred in the 1950s and 1960s, for Pakistanis the 1960s and 1970s, Bangladeshis the 1980s, and Black Africans the 1990s (Kelly et al. 2009). Similar to the United States, many complex factors continue to influence socioeconomic status, racial/ethnic relations, and cultural integration that vary significantly across different minority ethnic groups, which could impact racial/ethnic differences in adolescent well-being. For example, Indian people in the United Kingdom have achieved similar socioeconomic and occupational status to Whites and command greater power over institutional resources, whereas Black Caribbean, Pakistani, and Bangladeshi people remain underrepresented in higher occupational classes (Phillips 1998).

### 1.3 Importance of early adolescence

Insights from a life-course perspective inform our understanding of the saliency of adolescent well-being; health outcomes in adolescence have a persistent effect on later life health (Sawyer et al. 2012). Adolescents are faced with increased development demands through changes in biological and social transitions. Early adolescence, which starts at nearly 11 years is an important, and understudied period of the life course, when increased prevalence of mood disorders has been observed (Merikangas et al. 2010). Scholars identify early adolescence as a sensitive phase due to the onset of puberty and the accompanying academic transitions, changes in academic expectations, and increasing influence of peer groups (Sawyer et al. 2012). Socioemotional health is an integral component of adolescent health (Costello et al. 2002) and has been linked to academic achievement, subsequent anxiety and depression, and eating pathology (Bartels et al. 2013; Bos et al. 2006). The importance of health and health inequalities in early adolescence is underscored in studies showing that mental health in childhood is linked with lower future test scores, welfare receipt, and schooling attainment (Currie and Stabile 2006), and socioemotional difficulties are consequential for a number of outcomes in adulthood, including educational attainment, employment, and health inequalities (Duncan and Brooks-Gunn 1997; Palloni 2006).

One of the critical tasks of adolescence is identity development (Erikson 1994), and more specifically, racial/ethnic identity development (Peck et al. 2014). Children in early adolescence face the additional burden of navigating racial/ethnic identities in a societal context of increased stigma and discrimination (Benner et al. 2018). Given that primary social influences move from parents to peers (Brown and Larson 2009), adolescents may encounter more racial/ethnic biases outside the home (Eccles et al. 1993). Indeed racial/ethnic relations become more consequential during adolescence as racial/ethnic minorities may face differential treatment and engage in more dialogue around race/ethnicity with family members and friends (Tatum 2003). At the same time, there is potential for conflicting cultural values with peers and family members, and this in turn may lead to family and peer conflict and poor socioemotional and cognitive development (McLaughlin, Hilt, and Nolen-Hoeksema 2007).

### 1.4 Current study

In the current study we seek to extend the extant literature by focusing on racial/ethnic differences in socioemotional and cognitive development in two large cohort studies in the United Kingdom and United States during early adolescence. Few studies have attended to this time in the life course, and even fewer have focused on cognitive and health domains and across developmental contexts (i.e., United States versus United Kingdom). In addition, we seek to more comprehensively examine potential racial/ethnic differences – within and between countries – by considering a host of adolescent and family characteristics that might also explain differences in early adolescent development. Previous comparative studies are limited by aggregating race/ethnic groups into a catch-all children of immigrant group, which obscures heterogeneity in socioeconomic, migratory, and health profiles (Modood 2003). Our conceptual model considers that observed racial/ethnic inequalities in outcomes may be due to the level and type of family resources to which adolescents are exposed; these resources span adolescent characteristics and family sociodemographic, cultural, and psychosocial domains that are known individual correlates of adolescent socioemotional well-being and cognitive development (Bianchi 2000; Heiland and Liu 2006; Mollborn 2016).

We hypothesize that Pakistani, Bangladeshi, and Black African and Caribbean groups in the United Kingdom and Black and Latino groups in the United States may be subjected to poorer socioemotional and cognitive adjustment during early adolescence. Prior literature on these racial/ethnic minority groups suggests measurable characteristics such as socioeconomic conditions and cultural factors may explain differences. In contrast, we hypothesize that Asian Americans in the United States and Indians in the United Kingdom may fare better, being viewed as ‘model minority’ groups. In line with evidence on the advantaged economic conditions of Asian Americans and Indians, we hypothesize sociodemographic characteristics to explain differences between these groups and their White peers. We also hypothesize that any inequalities observed between racial/ethnic minorities and their White peers will be more similar across the United States and United Kingdom when we control for differences in adolescent and family characteristics. Any remaining cross-national differences, after adjusting for adolescent and family differences, may suggest evidence of unmeasured resources. These resources could be related to social policies within each context or access to high quality social support, or to other unmeasured dimensions we may expect to influence adolescent development, such as discrimination and racial microaggressions.

## 2. Data

To achieve our innovative comparative examination of race/ethnic inequalities in early adolescent development in the United Kingdom and the United States, we use the UK Millennium Cohort Study (MCS) and the US Early Childhood Longitudinal Study – Kindergarten Cohort Study (ECLS-K), which have comparable parallels in historical timing and design. The MCS is a nationally representative sample of 19,244 children born in the United Kingdom between 2000 and 2002, who were living in the United Kingdom at 9 months old, and who were eligible to receive Child Benefit (a universal child cash welfare benefit) (Plewis et al. 2004). The sample is clustered at the electoral ward level, and disadvantaged residential areas and those with a high proportion of ethnic minority residents were oversampled. Primary respondents were mothers (98%), and data collection occurred when children were 9 months, and 3, 5, 7, and 11 years old. Interview data was available for 69% of families when cohort members were aged 11. During home interviews, questions addressed child socioemotional development, socioeconomic circumstances, cultural tradition factors, and the psychosocial environment. At the fifth wave (age 11), trained interviewers carried out cognitive assessments and questions were asked to the cohort members about their self-esteem. We focused on the fifth data wave because the age at follow-up was the most comparable with the US kindergarten sample (see Plewis et al. 2004 for more details about sampling weights and survey methods employed).

The ECLS-K is a nationally representative, longitudinal study of approximately 21,400 kindergarteners’ educational experiences and development from kindergarten to 8^th^ grade. The data were collected on children who entered kindergarten in both public and private schools in the fall of 1998. The study used a dual-frame multistage sampling design. Initially, 100 primary sampling units (typically counties) were selected across the United States, after which 1,000 schools with kindergartens within these units were selected. Approximately 23 students from each school were then randomly selected. Data (i.e., parent, child, teacher, and school administrator surveys, direct child assessments) was collected in the fall and spring of kindergarten and 1^st^ grade and in the spring of 3^rd^, 5^th^, and 8^th^ grades. Approximately 75% of the sample maintained their participation throughout each data collection wave. Due to planned sample attrition, the ECLS-K did not follow approximately 8,500 children who moved schools between kindergarten and 5^th^ grade (Tourangeau et al. 2009). In the ECLS-K, 94% of children sampled in the base year (kindergarten) who did not move completed child assessments in 5^th^ grade, and 91% of those children had completed 5^th^ grade parent interviews (unweighted percentages; see Tourangeau, Lê, and Nord 2005 for description of completion rates). Our analyses focus on data from wave 6 when children were, on average, 11.2 years old (spring of 5^th^ grade).

An important challenge in comparative research is the availability of comparable data. Although the children in the MCS and ECLS-K differ in nearly 8 years in birthdates, we are fortunate to use contemporary large-scale data with representative samples of racial/ethnic groups and detailed sociodemographic variables. One outcome – vocabulary scores and academic performance from the cognitive domain – is fully comparable across the two countries. Although the other outcome – scores from the socioemotional domain – is comparable in measurement the reporting differs between the two countries (maternal report in MCS and teacher report in ECLS-K). Despite this difference in informant, assessing racial/ethnic patterning in socioemotional development gives an indication of the extent to which observed inequalities can be generalized to other areas of development. Additionally, it should be noted that the vast majority of the ECLS-K child sample (2%) was not foreign born, thus suggesting comparability in terms of child nativity between the US and UK datasets (100% of the MCS child sample were native born). The percent missing on covariates ranged from 0%–10% in the MCS and 0%-1% in the ECLS-K.

### 2.1 Measures

#### 2.1.1 Socioemotional development

In the MCS, during the interview, parents answered questions about their child’s socioemotional behavior using the Strengths and Difficulties Questionnaire (SDQ), age 4–15 year version (http://www.sdqinfo.com/). Strengths of the SDQ are its psychometric properties, representation of children’s socioemotional strengths and difficulties (Goodman 2001), and its wide use in research examining ethnic inequalities. The questionnaire asks five questions in each of five domains: emotional symptoms, conduct problems, hyperactivity–inattention, peer problems, and prosocial behavior. Each question is scored 0 (not at all true), 1 (partly true), or 2 (certainly true), with some questions reverse coded. Scores are summed across the first four domains to construct a total difficulties score, which was analyzed as a continuous variable with higher values indicating increased difficulties. An externalizing behavior score was created using scores from the conduct problems and hyperactivity–inattention scales, and an internalizing behavior score was created by summing emotional symptoms and peer problems scores (*α* = .89 and .77 for externalizing and internalizing behaviors, respectively). In descriptive analyses we used raw scores, and in regressions socioemotional development was standardized to have a mean of 0 and a standard deviation of 1 for easy interpretation and comparability to the ECLS-K.

In the ECLS-K, data on socioemotional development was collected from teachers’ responses on the internalizing and externalizing behavior subscales of the Social Rating Scale (SRS), a modified version of Gresham and Elliott’s (1990) Social Skills Rating System (Pollack et al. 2005). Externalizing behaviors assessed the frequency that a child argued, fought, got angry, acted impulsively, and disturbed ongoing activities. Internalizing behaviors assessed how frequently children appeared to be anxious, lonely, and sad, and to have low self-esteem. Teachers were asked to evaluate the frequency of children’s internalizing and externalizing behaviors according to the following scale: 1 = never (a student never exhibits this behavior), 2 = sometimes (a student exhibits this behavior occasionally or sometimes), 3 = often (a student exhibits this behavior regularly but not all the time), 4 = very often (a student exhibits this behavior most of the time, and N/O = no opportunity (no opportunity to observe this behavior). For each scale, scores were averaged, with higher scores representing more difficulties (α = .89 and .77 for teacher-rated internalizing and externalizing behaviors, respectively). A total difficulties scale was assessed by summing externalizing and internalizing scales. Akin to the UK data, we used raw scores in descriptive tables and standardized scores for regressions to allow for comparability.

#### 2.1.2 Cognitive development: Verbal skills/academic performance

In the MCS, verbal skills were assessed using a subset of the British Ability Scales II (BAS II), which is a battery of cognitive abilities and educational achievement tests suitable for use from ages 2 years 6 months to 17 years 11 months (Elliott, Smith, and McCulloch 1997; Hill 2005). The individual subscales are widely validated, age appropriate, can be analyzed separately, and have been shown to predict later child cognitive performance (Jones and Schoon 2008). Data at age 11 used the Verbal Similarities subscale assessing children’s verbal reasoning and verbal knowledge (Hansen 2014). The subscale scores used in this study are standardized to mean 50 and standard deviation 10 and are adjusted for both item difficulty and age. Age adjustment is made within three-month age bands; thus we control for age at interview in our models to account for heterogeneity of verbal skills of children within these age bands.

Adolescent academic performance in the ECLS-K was assessed using direct assessments. At each data collection wave, children completed untimed, individually administered, two-stage standardized assessments in reading (e.g., vocabulary knowledge, reading comprehension). Adolescents’ performance on the uniform first stage determined whether they took the low-, medium-, or high-difficulty version of the second stage. Item Response Theory was used to develop single proficiency scores across stages (*M* = 142.91, *SD* = 21.93 for the current sample); these scores are directly comparable across years, as they represent specific points along the same ability continuum (Reardon 2005).

For both the MCS and ECLS-K, we used the raw cognitive development scores for descriptive tables and used standardized outcomes for comparability across countries. Higher scores reflect better functioning.

#### 2.1.3 Race and ethnicity

Racial and ethnic categories were constructed using mother reports of the child, based on categories derived from country-specific census data. For the United Kingdom, groups were: White, Indian, Pakistani, Bangladeshi, Black Caribbean (including mixed White and Black Caribbean), Black African (including mixed White and Black African), and Other (mixed racial/ethnic groups and racial/ethnic minority groups that could not be categorized into any of the other groups). For the ECLS-K, categories were: White, Asian American, Black, Latino, and Other.

It is important to note that we rely on MCS and ECLS-K categorizations that are in line with country-specific census classifications. In the ECLS-K, we are unable to make distinctions among different subgroups within each large, race/ethnic group (e.g., Mexican, Puerto Rican, or Dominican, among those in the Hispanic category) due to data unavailability (mothers were simply asked whether they were of Hispanic/Latina origin). Further, we do not make distinctions on the basis of recency of parental immigration, citizenship status, or whether arrival in host countries was voluntary or involuntary, factors with which race, ethnicity, social class, and income are often confounded (Coll et al. 1996). These are compelling dimensions to examine but are outside the scope of our research questions, particularly so as there is little comparable data on these factors. Nevertheless, we adjust for parental nativity in order to account for possible differences between children born to migrant and non-migrant parents.

#### 2.1.4 Covariates

We examined the contribution of adolescent and family resources that could account for potential racial/ethnic inequalities in early adolescent development across sociodemographic, cultural, and psychosocial domains. Covariates were assessed concurrently to early adolescent outcomes with the exception of mother’s age at birth, which we consider as a background control variable. In both datasets, we assessed adolescent control variables using age in years and gender (female is reference). Sociodemographic covariates were: equivalized household income in quintiles (middle income quintile as reference), highest parental education, single parenthood (two parent family as reference), mother employment (on leave/not working as reference), and mother’s age at birth. Note that while measures of education are meaningful in each country and broadly comparable, the categories used were necessarily different (for more details, see Teitler et al. 2007). Cultural tradition covariates were: binary indicators of English as primary language spoken at home and whether mother was foreign born (Kelly, Watt, and Nazroo 2006; Zilanawala et al. 2015a). A marker of the psychosocial environment assessed maternal mental health. In MCS, maternal mental health was assessed via the K6, a screening scale for assessing psychological distress (α = 89) (Kessler et al. 2002). In the ECLS-K, a similar construct was assessed using 12 items from the Center for Epidemiologic Studies Depression Scale (CES-D) (α = .89) (Radloff 1977).

### 2.2 Analytic sample

Adolescent socioemotional and cognitive development is influenced by multiple births, and therefore we analyzed data on singleton-born adolescents for whom mothers reported on their child’s race/ethnicity and child socioemotional health. The analytic sample was 10,188 in MCS and 6,950 in ECLS-K after selecting on observations complete on covariates. Note that the ECLS-K sample size is rounded to the nearest ten throughout per NCES requirements for restricted-use data. Analytic samples varied slightly by the outcome measure (see Table 1 for more details).

### 2.3 Planned analyses

We begin by presenting descriptive data on adolescents by racial and ethnic groups. For our primary analysis, we used estimated Ordinary Least Square regressions to examine the contribution of child race/ethnicity to early adolescent development beyond the effects of family resources. In the baseline model (Model 1), we examined racial/ethnic inequalities controlling for adolescent age and gender. Next, we separately adjusted for the three domains of covariates of family resources across a series of models: Model 2 adjusted for adolescent control variables and family sociodemographic resources; Model 3 adjusted for adolescent control variables and cultural tradition resources; Model 4 adjusted for adolescent control variables and maternal mental health. A final, fully adjusted model (Model 5) included all adolescent control variables and family resource covariates. All models used the majority group (White adolescents) as the reference category. All analyses use sample weights to adjust for unequal probability of being sampled and the stratified and clustered sample design. As with other comparative studies (Nazroo et al. 2018; Washbrook et al. 2012; Zilanawala et al. 2015a), we can use the standardized developmental outcomes of the average adolescent in each country as a benchmark relative to which their ethnic minority peers in each country can be assessed.

## 3. Results

### 3.1 Race/ethnic variation in outcomes and explanatory factors

Tables 1 and 2 present mean inequalities in early adolescent cognitive and socioemotional development and explanatory factors by race/ethnicity subgroup for the United Kingdom and United States, respectively. In the United Kingdom, nearly 13% of adolescents were from a minority racial/ethnic group whereas this was true for 35%–38% of adolescents in the United States. The largest racial/ethnic minority group in the United Kingdom was Pakistani (3.4%) and in the United States was Latino (15%–16%). Racial/ethnic inequalities were found in both national settings. For adolescent outcomes, in the United Kingdom, Pakistani and Black Caribbean 11 year olds had disadvantages in socioemotional outcomes and verbal skills: both groups had higher total difficulties, externalizing, and internalizing scores and the lowest verbal scores. Indian and Black African adolescents had the lowest scores on socioemotional outcomes. Indian adolescents had the highest verbal skills scores. In the United States, Asian American adolescents had the lowest scores on total difficulties, externalizing, and internalizing scales, whereas Black and Latino adolescents had the highest total difficulties and externalizing scores. Black and Latino adolescents had the lowest verbal scores.

For the sociodemographic factors, stark racial/ethnic inequalities were also evident for both countries. Pakistani, Bangladeshi, Black Caribbean, and Black African adolescents in the United Kingdom and Asian American, Black, and Latino adolescents in the United States were most likely to be living in households with annual household incomes in the lowest quintile. In the United Kingdom, mothers of Pakistani and Bangladeshi adolescents had the lowest educational attainment, and this was true for mothers of Black and Latino adolescents in the United States as well. Mothers of Pakistani and Bangladeshi adolescents had the lowest employment rates in the United Kingdom, and similar patterns were observed for mothers of Latino adolescents in the United States.

In relation to cultural tradition factors, Pakistani and Bangladeshi adolescents in the United Kingdom were most likely to live in a household in which the primary language spoken was not English. For psychosocial factors, in the United Kingdom, there was little variation in maternal mental health scores across race/ethnicity; in the United States, mothers of Black and Latino adolescents had the poorest mental health.

### 3.2 The effects of adolescent characteristics and sociodemographic, cultural, and psychosocial resources for UK sample

Table 3 illustrates the relations between mean standardized scores of early adolescent development, race/ethnicity, and a range of explanatory factors in the United Kingdom. Panel A presents results for total difficulties scores. Higher scores indicate more behavioral problems.

We found Indian and Black African adolescents to have nearly one-fifth of a standard deviation lower child behavior scores at age 11 compared to their White peers (Model 1, Panel A). Conversely, Pakistani and Black Caribbean adolescents had more problem behaviors (.16–.20 standard deviation, Model 1, Panel A). The levels of family resources explaining these gaps varied by racial/ethnic group. Sociodemographic factors played a large role in explaining the lower total difficulties scores for Indian adolescents and the higher scores for Pakistani and Black Caribbean adolescents versus White youth (Model 2). After adjusting for the relative economic disadvantage of families of Bangladeshi adolescents, they had fewer problems behaviors at age 11 compared with their White peers. Cultural tradition factors also partially explained differences for Indian and Black African adolescents, whereas maternal mental health helped explain inequalities for Pakistani, Bangladeshi, and Black Caribbean adolescents (Models 3 and 4). Lastly, in the fully adjusted model (Model 5) which captured adolescent and family resources from our conceptual model, we found Black African adolescents to have significantly lower total difficulties scores (.25 standard deviation) compared to White adolescents. It also appears that adjusting for the relatively adverse family resources of Pakistani and Bangladeshi adolescents disguises an advantage in problem behaviors (.12 and .24 standard deviation, respectively).

The results for externalizing and internalizing behaviors are summarized in Panels B and C and show a similar set of patterns to Panel A. Generally, we see Indian youth to have lower externalizing and internalizing behaviors and these advantages, relative to White youth, are attenuated by socioeconomic and cultural family resources. Both Bangladeshi and Pakistani adolescents experience advantages in externalizing behaviors (.19 and .38 standard deviation, respectively, Model 5, Panel B), after adjusting for family resources. However, both groups of adolescents experience a shortfall in internalizing scores (Panel B), which are largely accounted for when adjusting for socioeconomic characteristics and maternal mental health. Similar to their lower total difficulties scores, Black African adolescents have one-quarter and one-fifth of a standard deviation difference in externalizing and internalizing scores, net of average family characteristics.

Panel D considers verbal scores and higher scores reflect better functioning. We find consistent advantages for Indian children (Models 1–4); after adjusting for advantageous family resources, they perform better than their White peers (.30 standard deviation, Model 5). The opposite is true for Bangladeshi and Pakistani adolescents who had significantly lower verbal scores versus White youth (Model 1) and socioeconomic factors (Model 2) explained these inequalities.

### 3.3 The effects of adolescent characteristics and sociodemographic, cultural, and psychosocial resources for US sample

The corresponding results for US adolescents are illustrated in Table 4. Panel A reports racial/ethnic inequalities in total difficulties. The results for the United States are more straightforward compared to UK results: there is consistency in sign and significance across models and there is less variation in explanatory factors in understanding the gaps in development. Asian American adolescents have fewer problem behaviors compared to their White peers (.27 standard deviation, Model 1), and this advantage was partially explained by cultural tradition resources (Model 3). These differences were also true for the subdomains of externalizing and internalizing behaviors in Panels B and C. Black adolescents, in contrast, had more problem behaviors (Model 1), and this inequality was primarily accounted for by their relative economic disadvantage (Model 2). In fully adjusted models for externalizing and internalizing behaviors, the gap between Black and White adolescents was between one-fifth and nearly one-third of a standard deviation (Model 5 in Panels B and C).

Panel D examines racial/ethnic inequalities in verbal skills. In unconditional estimates, Black and Latino adolescents had significantly lower verbal scores in 5^th^ grade; nearly four-fifths and three-fifths of a standard deviation below the mean, respectively (Model 1). Models adjusting for socioeconomic resources reduce these gaps in half (Model 2) and estimates remain statistically significant in fully adjusted models (Model 5).

## 4. Discussion

The objective of this comparative study was to examine factors of the family environment that may explain possible racial/ethnic inequalities in an understudied population (i.e., early adolescents) in the United States and in the United Kingdom. A strength of our study is the use of a detailed race/ethnic classification in the investigation of racial/ethnic inequalities across two countries with different composition of racial/ethnic groups, migration patterns, social welfare systems, and racial/ethnic relations. Unlike previous comparative work (Washbrook et al. 2012), we find stronger differences between countries than we do across adolescent outcomes. In particular, poor socioemotional health and cognitive development disadvantages were associated with minority status in both countries, but the factors that explain the race/ethnic inequalities appear to differ between the United States and the United Kingdom.

In the United States, we found that cultural resources and family socioeconomic capital played a large role in attenuating differences in problem behaviors between Asian American, Black, and Latino adolescents and their White peers. In the United Kingdom, the explanatory factors explaining advantages and disadvantages in problem behaviors varied by racial/ethnic group. Sociodemographic factors played a large role in explaining the lower total difficulties scores for Indian adolescents and the higher scores for Pakistani and Black Caribbean adolescents versus White youth. Cultural tradition factors also partially explained differences for Indian and Black African adolescents, whereas maternal mental health helped explain inequalities for Pakistani, Bangladeshi, and Black Caribbean adolescents. On the one hand in both societies, there are strong family resource effects, suggesting that relative family advantages and disadvantages do have meaningful influences on adolescent socioemotional development and that levels of resources do explain cross-national differences. Additionally, there is suggestive evidence that the broader range of family background variables and the variation of these resources by race/ethnicity in the United Kingdom means that families may matter more in the United Kingdom than in the United States. This counters Esping-Andersen et al. (2002) thesis that a more developed welfare state in the United Kingdom can substitute for family resources. On the other hand family resources had very little influence on the advantages seen for Black African adolescents who had nearly a quarter standard deviation difference in their problem behavior scores compared to White adolescents in fully adjusted models. Equally, there are a myriad of unobserved family environment factors which may further explain racial/ethnic differences observed here. Perhaps public policies or local government investments allow some parents to enhance their investment in their children by adjusting their family capital in ways that are beneficial to their socioemotional development, a hypothesis that warrants additional research.

Turning to verbal skills, in both contexts we find family resources cannot explain the sizable cross-country differences. In the United Kingdom Indian adolescents have nearly one-third of a standard deviation increase in their verbal scores, an advantage that is unaffected by levels of family resources (fully adjusted models). Evidence elsewhere suggests that Indian children’s high verbal performance originates in early childhood and is promoted both by family socioeconomic characteristics and cognitively stimulating home environments in early childhood (Zilanawala, Kelly, and Sacker 2016). In the United States Black and Latino adolescents had significantly lower verbal scores; nearly two-fifths and one-fifth of a standard deviation below the mean, respectively. Although socioeconomic conditions did reduce unadjusted estimates by one-half. The strong economic effects in the US context could be due to the greater income inequality in the United States than in the United Kingdom (Smeeding 2005). Overall, results from both societies suggest effect sizes in racial/ethnic group differences in adolescent development are similar and there is a strong role of family resources in explaining differences by race/ethnicity. Where there are remaining inequalities or advantages in development, in our final models, we can speculate that differences reflect unmeasurable features of each context that may promote or hinder development of racial/ethnic minority groups. This is a critical research question, particularly when the implications of adolescent development on future outcomes are considered.

Although our findings on race/ethnic differences in the United Kingdom have some similarities to previous studies, we also find some interesting contrasts. That we find initial disadvantages in socioemotional health among Pakistani and Black Caribbean adolescents is distinct to other studies examining adolescents (Astell-Burt et al. 2012; Fagg et al. 2006; Maynard, Harding, and Minnis 2007). However, we find fewer behavioral problems among Indian, Black African, and Bangladeshi adolescents in the fully adjusted models; results that corroborate other findings among British adolescents (Maynard, Harding, and Minnis 2007; Stansfeld et al. 2004). Marked differences between previous studies and the current analyses make it difficult to compare findings in the United Kingdom. First, prior work has used wide age ranges, such as 5 to 16 years of age (Goodman, Patel, and Leon 2010) or 11 to 16 years of age (Astell-Burt et al. 2012). Second, studies finding evidence of fewer socioemotional difficulties among minority adolescents use contextually specific data, for example, data on children in select London schools (Astell-Burt et al. 2012) or in specific English cities (Dogra et al. 2013), thus making it difficult to extrapolate such findings to the general population. Third, many other studies have used adolescent self-reports of socioemotional difficulties (Maynard, Harding, and Minnis 2007; Stansfeld et al. 2004), while we incorporated mother reports. The factors representing socioeconomic conditions, cultural tradition, and maternal psychosocial circumstances investigated in the present study may operate differently across different years in adolescence, and we have focused on a very particular time period (i.e., early adolescence). Future research should examine the longitudinal trajectory of socioemotional difficulties during early through late adolescence and potential variations in reports of socioemotional difficulties by mothers, teachers, and children.

A venerable literature has been developed on inequalities in markers of child health in the US context (Magnuson, Lahaie, and Waldfogel 2006; Magnuson and Waldfogel 2005). However, we focus on 5^th^ graders in isolation, which is a clear difference with previous studies (Cheng and Powell 2005; Crosnoe 2007; Galindo and Fuller 2010), making it difficult to compare findings in our US analyses to the extant literature that generally focuses on early childhood development. That we find socioemotional advantages for Asian American adolescents and disadvantages for Blacks, along with significantly lower verbal scores for Blacks and Latinos is in line with prior literature focusing on early childhood (Han, Lee, and Waldfogel 2012). Findings from one comparative study aggregating race/ethnic minority children in a catch-all ‘immigrant children group’ suggests lower verbal performance among children in the United States who were born to foreign-born mothers; however this study examines 5-year-old children (Washbrook et al. 2012).

The literature on the link between family socioeconomic conditions and children’s mental health is decidedly mixed. There is evidence that disadvantaged socioeconomic status is associated with more socioemotional difficulties, using data on children of mixed age ranges (Costello et al. 2003) or focusing on early childhood (Duncan and Brooks-Gunn 1997), whereas some studies do not report any significant association between material disadvantage and socioemotional difficulties among adolescents (Fagg et al. 2006; Maynard, Harding, and Minnis 2007). One study finds fewer socioemotional difficulties among Bangladeshi adolescents in the United Kingdom, despite being the most economically disadvantaged (Stansfeld et al. 2004). One explanation for the incongruent findings on socioeconomic circumstances is that compared with childhood (and adulthood), adolescence is a period of relative health equality (West 1997), and it is hypothesized that school, peer influences, and youth culture overcome family effects on health (West, Sweeting, and Leyland 2004). Studies using multiple indices of socioeconomic circumstances corroborate our results (Amone-P’Olak et al. 2009; Davis et al. 2010). However, our results also indicate that socioeconomic circumstances do not explain the advantages and disadvantages in our sample for race/ethnic minority children in the United States and United Kingdom (e.g., fewer problem behaviors for Black African adolescents in the United Kingdom, and lower verbal scores for Black and Latino American adolescents) even after considering multiple measures of family’s economic conditions.

We emphasize that the goal of our study was to document the extent of racial/ethnic inequalities in early adolescent development across the United Kingdom and United States, taking other known contributing factors into account. We briefly discuss three important implications of our work. First, studies on adolescent development that exclusively investigate one marker of health and development may present a distorted conclusion on racial/ethnic patterning of development, which preferably considers a range of abilities (socioemotional and cognition). Second, we find some advantages in the socioemotional domain for Indian youth in the United Kingdom and Asian American adolescents in the United States, giving some support for the ‘model minority’ hypothesis. The risk of applying a minority model construct in the United Kingdom and United States is that the opposite, or a ‘deficit model,’ can be inferred for other ethnic minority adolescents who may be perceived as underachieving or having poor behavior (Wong 2015). This could potentially reinforce negative stereotypes about the behavioral or cognitive skills of Black African, Pakistani, and Bangladeshi youth in the United Kingdom or African American and Hispanic adolescents in the United States. Moreover, such model minority labels also presume homogeneity in the Asian American population, yet prior research documents vast differences in developmental outcomes across specific Asian ethnic groups in the United States (Lee 2015). Third, we do find racial/ethnic differences in socioemotional and cognitive development across the two countries after including adolescent and family resources. One possible interpretation is that these unexplained differences may relate to public policy, school systems, or integration policies within each country context that may promote or obstruct gaps in adolescent development. Of course, it cannot be ruled out that unobservable differences between race/ethnic minority groups may exist that were not captured in the current study. It is an important priority for future comparative research to carefully disentangle the policies that do or do not support the success of racial/ethnic minority adolescents’ well-being.

A strength of this study is that we examined data on objective measures, collected by trained observers, of verbal ability in children. It is noteworthy that we used teacher and mother reports of socioemotional skills in the United States and United Kingdom, respectively. Indeed there is evidence of systematic variation in reporting of socioemotional behaviors between teachers and mothers’ by race/ethnicity (Miner and Clarke-Stewart 2008). It is a challenge in comparative research to find comparable data without sacrificing detail. To be sure, our findings lend themselves to generalizing to their respective populations. Future data collection efforts could focus on measurement equivalence and informant discrepancies across countries. Our cultural tradition markers were limited. For example, the marker of language spoken at home does not allow us to disentangle the benefits of a bilingual home environment, such as cognitive flexibility and positive cultural capital, from the disadvantages in language expression resulting from exposure to a non-English-speaking home environment. Future comparative data analyses should consider detailed questions on English language use, both within the household and with friends. Another limitation is the diversity within each race/ethnic group. Our study did not consider the differences in cultural backgrounds of various subgroups (e.g., Chinese, Filipinos, Puerto-Ricans, Arabs) that may influence the generalizability of the data. Additionally, with the exception of mother’s age at birth, we used a cross-sectional design, which precludes the temporal ordering of other domains that could influence development prior to adolescence (e.g., maternal employment and low family income) and changes in inequalities over time. Future studies are needed to examine the trajectories of racial/ethnic patterning over time and their long-term implications in relation to adolescent development.

In an age of ever increasing global migration and economic inequalities, it has become salient to understand race/ethnic inequalities faced by adolescents, particularly during early adolescence, an important life-course period predicting future earnings and occupational attainment (Claessens, Duncan, and Engel 2009). We were able to harness comparable US and UK cohort data to show that marked inequalities in socioemotional health and development exist among 11 year olds. Our findings underscore work emphasizing the significance of incorporating the full range of health (behavioral and cognitive) to avoid inaccurate conclusions of race/ethnic patterning between the United States and United Kingdom, or other comparative analyses (Washbrook et al. 2012). Our results also raise the question of how adolescents navigate school systems and their future labor force participation and romantic relationships given inequalities in socioemotional and cognitive domains present in adolescence. Understanding the developmental trajectories of racial/ethnic minorities across the life course is an important topic of future research so that we can better evaluate public policy and educational systems in supporting or failing these adolescents.

## Figures and Tables

**Table 1:** Early adolescent development and explanatory factors by race/ethnicity: United Kingdom (mean or percent, (SD))

	White (n = 8910)	Indian (n = 242)	Pakistani (n = 349)	Bangladeshi (n = 113)	Black Caribbean (n = 204)	Black African (n = 165)	Other (n = 203)
**Outcomes**							
Total difficulties	7.78 (5.95)	6.90 (5.81)	8.71 (6.06)	7.82 (6.96)	9.00 (5.70)	6.66 (4.68)	7.84 (5.41)
Externalizing	4.58 (3.63)	4.11 (3.57)	4.74 (3.65)	3.91 (4.34)	5.28 (3.62)	3.76 (2.83)	4.26 (3.14)
Internalizing	3.20 (3.22)	2.79 (3.15)	3.96 (3.32)	3.91 (3.68)	3.73 (2.95)	2.92 (2.51)	3.57 (3.22)
Verbal scores	58.72 (9.54)	62.33 (10.08)	55.59 (11.59)	54.51 (14.94)	57.47 (9.57)	59.78 (7.69)	59.61 (8.95)
**Sociodemographic factors**							
Equivalized household income (quintiles)							
Bottom	14.5	8.0	62.6	71.5	31.5	25.8	22.2
Second	19.2	25.6	26.1	14.3	30.1	29.9	15.3
Third	20.5	27.0	5.7	13.3	16.7	21.0	22.7
Fourth	22.8	18.3	2.7	1.0	12.9	8.6	14.7
Top	23.0	21.0	2.9	0.0	8.8	14.7	25.1
Highest educational qualification in household							
None	11.3	6.6	30.4	32.0	12.7	14.4	16.4
Overseas qualifications only	1.7	8.2	11.5	8.3	3.4	15.6	6.6
NVQ1	6.5	13.9	7.1	3.5	7.6	13.5	12.2
NVQ2	27.6	28.6	12.3	12.1	28.4	33.4	31.4
NVQ3	8.8	15.3	7.2	6.0	5.4	4.9	9.3
NVQ4	32.7	22.2	24.0	30.5	34.7	14.2	20.1
NVQ5	11.5	5.2	7.6	7.6	7.9	4.0	3.9
Family structure							
Two parents	77.4	90.8	84.7	88.9	42.2	57.8	70.5
One parent	22.6	9.3	15.4	11.1	57.8	42.2	29.5
Mother’s employment							
In work/leave	71.5	77.3	31.8	32.2	61.3	60.7	62.2
Not in work/leave	28.5	22.7	68.2	67.8	38.7	39.3	37.9
Mother’s age at birth	28.53 (5.92)	28.63 (5.21)	25.91 (5.85)	25.35 (4.71)	27.80 (5.74)	30.17 (5.67)	29.32 (5.75)
**Cultural tradition factors**							
Language spoken at home							
Mostly English	99.4	75.9	65.7	46.2	96.9	81.9	75.2
Mostly other	0.6	24.1	34.3	53.8	3.1	18.1	24.8
Mother’s generational status, %							
Born in UK	96.3	53.7	48.8	11.4	90.2	44.1	48.3
Foreign born	3.7	46.3	51.2	88.7	9.8	55.9	51.7
**Psychosocial factor**							
Mother’s mental health	25.99 (4.46)	25.36 (5.07)	24.15 (5.52)	23.87 (6.78)	24.91 (4.04)	25.35 (4.34)	25.43 (4.03)

*Note*: Sample excludes twins and triplets. Standard deviations are presented in parentheses. Sample size is 10,188 but varies slightly for some outcome measures because of differential response. Outcomes are presented in raw scores and standardized for regressions. All means are weighted by sample weights. Sample sizes are unweighted.

**Table 2:** Early adolescent development and explanatory factors by race/ethnicity: United States (mean or percent, (SD))

	White	Asian American	Black	Latino	Other
**Outcomes**					
Total difficulties	3.19 (0.87)	2.94 (0.80)	3.43 (0.92)	3.23 (0.89)	3.24 (0.83)
Externalizing	1.58 (0.53)	1.44 (0.51)	1.83 (0.65)	1.61 (0.56)	1.62 (0.52)
Internalizing	1.61 (0.54)	1.50 (0.44)	1.61 (0.50)	1.62 (0.53)	1.62 (0.50)
Verbal scores	147.16 (19.94)	144.67 (20.87)	128.70 (23.10)	133.74 (22.38)	138.25 (24.98)
**Sociodemographic factors**					
Equivalized household income (quintiles)					
Bottom	7.9	17.5	41.1	33.6	23.8
Second	16.7	22.6	26.9	28.4	20.2
Third	22.2	19.0	16.5	16.7	19.5
Fourth	23.9	14.1	8.1	12.2	12.3
Top	29.3	26.7	7.4	9.1	24.2
Highest educational qualification in household					
Less than high school diploma/GED	1.5	4.4	10.4	18.1	4.5
High school diploma/GED	14.5	13.1	26.5	23.5	13.4
Vocational or technical program	4.5	5.4	7.9	7.1	4.1
Some college	28.9	21.7	34.6	27.9	37.1
Bachelor’s degree	25.5	28.3	12.7	12.7	19.9
Graduate school	25.1	27.1	7.9	10.7	21.0
Family structure					
Two parents	86.4	90.0	45.4	79.6	76.6
One parent	13.6	10.0	54.6	20.4	23.4
Mother’s employment					
In work/leave	78.8	77.8	77.1	68.7	71.1
Not in work/leave	21.2	22.2	22.9	31.3	28.9
Mother’s age at birth	25.77 (5.19)	25.95 (5.58)	21.52 (4.76)	22.84 (5.19)	23.76 (5.68)
**Cultural tradition factors**					
Language spoken at home					
Mostly English	99.9	96.6	99.7	97.4	99.3
Mostly other	0.1	3.4	0.3	2.6	0.7
Mother’s generational status					
Born in US	95.7	22.2	91.1	46.5	85.6
Foreign born	4.3	77.8	8.9	53.5	14.4
**Psychosocial factor**					
Mother’s mental health	1.33 (0.39)	1.37 (0.47)	1.50 (0.53)	1.52 (0.73)	1.36 (0.40)

*Note*: *N_range_* is 4,300–4,540 for Whites, 370–410 for Asian Americans, 540–580 for Blacks, 1,030–1130 for Latino, and 270–290 for other races/ethnicities. All *N*’s rounded to the nearest 10 per NCES requirements for restricted-use data. Outcomes are presented in raw scores and standardized for regressions.

**Table 3:** Multivariate linear regressions predicting racial/ethnic inequalities in early adolescent development: United Kingdom

	Model 1	Model 2	Model 3	Model 4	Model 5
	Coeff. (SE)	Coeff. (SE)	Coeff. (SE)	Coeff. (SE)	Coeff. (SE)
**Panel A: Total difficulties (N = 10,186)**					
Indian	−0.16 (0.07)*	−0.09 (0.07)	−0.12 (0.07)	−0.22 (0.06)***	−0.12 (0.07)
Pakistani	0.16 (0.05)***	−0.14 (0.06)*	0.20 (0.06)***	−0.01 (0.05)	−0.12 (0.05)*
Bangladeshi	0.02 (0.10)	−0.28 (0.10)**	0.10 (0.11)	−0.17 (0.08)*	−0.24 (0.09)**
Black Caribbean	0.20 (0.09)*	0.01 (0.09)	0.21 (0.09)*	0.10 (0.09)	0.00 (0.09)
Black African	−0.20 (0.09)*	−0.31 (0.09)***	−0.15 (0.09)	−0.26 (0.09)***	−0.25 (0.09)**
Other	0.01 (0.08)	−0.02 (0.08)	0.06 (0.08)	−0.04 (0.07)	0.02 (0.08)

**Panel B: Externalizing behaviors (N = 10,188)**					
Indian	−0.14 (0.07)*	−0.08 (0.08)	−0.08 (0.08)	−0.19 (0.07)**	−0.08 (0.08)
Pakistani	0.05 (0.05)	−0.23 (0.06)***	0.11 (0.06)*	−0.09 (0.05)	−0.19 (0.05)***
Bangladeshi	−0.16 (0.10)	−0.45 (0.10)***	−0.04 (0.11)	−0.32 (0.09)***	−0.38 (0.10)***
Black Caribbean	0.18 (0.09)	0.01 (0.10)	0.19 (0.09)*	0.10 (0.09)	0.01 (0.09)
Black African	−0.24 (0.08)***	−0.32 (0.09)***	−0.17 (0.08)*	−0.29 (0.09)***	−0.25 (0.09)**
Other	−0.09 (0.08)	−0.10 (0.08)	−0.02 (0.08)	−0.13 (0.07)	−0.05 (0.08)

**Panel C: Internalizing behaviors (N = 10,195)**					
Indian	−0.13 (0.07)*	−0.09 (0.06)	−0.13 (0.07)	−0.18 (0.06)***	−0.13 (0.06)*
Pakistani	0.24 (0.06)***	−0.00 (0.06)	0.24 (0.07)***	0.08 (0.05)	−0.01 (0.06)
Bangladeshi	0.22 (0.10)*	−0.00 (0.10)	0.23 (0.11)*	0.05 (0.09)	−0.01 (0.09)
Black Caribbean	−0.10 (0.09)	0.00 (0.09)	0.17 (0.08)*	0.07 (0.09)	−0.00 (0.09)
Black African	0.12 (0.09)	−0.22 (0.09)**	−0.10 (0.09)	−0.15 (0.08)	−0.18 (0.08)*
Other	0.37 (0.30)	0.08 (0.09)	0.13 (0.10)	0.07 (0.09)	0.09 (0.10)

**Panel D: Verbal scores (N = 10,033)**					
Indian	0.36 (0.06)***	0.30 (0.06)***	0.34 (0.06)***	0.38 (0.06)***	0.30 (0.06)***
Pakistani	−0.32 (0.12)**	−0.01 (0.10)	−0.31 (0.12)**	−0.30 (0.12)*	−0.01 (0.10)
Bangladeshi	−0.42 (0.18)*	−0.07 (0.16)	−0.43 (0.19)*	−0.35 (0.17)*	−0.09 (0.17)
Black Caribbean	−0.13 (0.10)	−0.00 (0.10)	−0.13 (0.10)	−0.09 (0.10)	−0.01 (0.10)
Black African	0.07 (0.08)	0.13 (0.07)	0.02 (0.08)	0.09 (0.07)	0.09 (0.08)
Other	0.09 (0.08)	0.09 (0.07)	0.06 (0.08)	0.10 (0.08)	0.07 (0.07)

*Note*:

* p<0.05,

** p<0.01,

*** p<0.001 for two-tailed significance tests.

Coefficients represent standard deviations of outcome. White is the reference group. Model 1 adjusts for child age and gender. Model 2 adjusts for Model 1 and for household income, highest educational qualification, family structure, maternal employment, and mother’s age at birth. Model 3 adjusts for Model 1 and for language spoken at home and mother’s nativity. Model 4 adjusts for Model 1 and for mother’s mental health. Model 5 adjusts for all covariates included in Models 1 to 4. All estimates are weighted with sample weights.

**Table 4:** Multivariate linear regressions predicting racial/ethnic inequalities in early adolescent development: United States

	Model 1	Model 2	Model 3	Model 4	Model 5
	Coeff. (SE)	Coeff. (SE)	Coeff. (SE)	Coeff. (SE)	Coeff. (SE)
**Panel A: Total difficulties (N = 6,500)**					
Asian American	−0.27 (0.05)***	−0.27 (0.05)***	−0.13 (0.06)*	−0.27 (0.05)***	−0.14 (0.06)*
Black	0.29 (0.05)***	0.05 (0.05)	0.30 (0.05)***	0.26 (0.05)***	0.05 (0.05)
Latino	0.05 (0.03)	−0.10 (0.04)**	0.13 (0.04)***	0.02 (0.03)	−0.02 (0.04)
Other	0.07 (0.06)	−0.01 (0.06)	0.09 (0.06)	0.06 (0.06)	0.00 (0.06)

**Panel B: Externalizing behaviors (N = 6,570)**					
Asian American	−0.24 (0.05)***	−0.23 (0.05)***	−0.10 (0.06)	−0.24 (0.05)***	−0.11 (0.06)
Black	0.47 (0.04)***	0.30 (0.05)***	0.48 (0.04)***	0.46 (0.04)***	0.30 (0.05)***
Latino	0.06 (0.03)	−0.04 (0.04)	0.15 (0.04)***	0.04 (0.03)	0.03 (0.04)
Other	0.09 (0.06)	0.03 (0.06)	0.11 (0.06)	0.09 (0.06)	0.04 (0.06)

**Panel C: Internalizing behaviors (N = 6,530)**					
Asian American	−0.20 (0.05)***	−0.22 (0.05)***	−0.13 (0.06)*	−0.21 (0.05)***	−0.13 (0.06)*
Black	−0.01 (0.05)	−0.22 (0.05)***	−0.00 (0.05)	−0.03 (0.05)	−0.22 (0.05)***
Latino	0.02 (0.04)	−0.12 (0.04)***	0.07 (0.04)	−0.01 (0.04)	−0.07 (0.04)
Other	0.02 (0.06)	−0.05 (0.06)	0.03 (0.06)	0.02 (0.06)	−0.04 (0.06)

**Panel D: Verbal scores (N = 6,950)**					
Asian American	−0.10 (0.05)*	−0.04 (0.05)	−0.04 (0.06)	−0.10 (0.05)	−0.04 (0.05)
Black	−0.84 (0.04)***	−0.41 (0.04)***	−0.83 (0.04)***	−0.80 (0.04)***	−0.41 (0.04)***
Latino	−0.61 (0.03)***	−0.21 (0.03)***	−0.57 (0.04)***	−0.57 (0.03)***	−0.20 (0.04)***
Other	−0.41 (0.06)***	−0.25 (0.05)***	−0.40 (0.06)***	−0.40 (0.06)***	−0.26 (0.05)***

*Note*:

* p<0.05,

** p<0.01,

*** p<0.001 for two-tailed significance tests.

Coefficients represent standardized outcome scores. White is the reference group. Model 1 adjusts for child age and gender. Model 2 adjusts for Model 1 and for household income, highest educational qualification, family structure, maternal employment, and mother’s age at birth. Model 3 adjusts for Model 1 and for language spoken at home and mother’s nativity. Model 4 adjusts for Model 1 and for mother’s mental health. Model 5 adjusts for all covariates included in Models 1 to 4. All *N*’s rounded to the nearest 10 per NCES requirements for restricted-use data.
